# The 1st Symposium of the Wellcome Trust-Funded Multi-Collaborative Microbial Pathogen Microarray Facility—BμG@S 2002: ‘Bacterial Pathogens, Microarrays and Functional Genomics’

**DOI:** 10.1002/cfg.189

**Published:** 2002-08

**Authors:** Philip D. Butcher

**Affiliations:** Department of Medical Microbiology St George's Hospital Medical School Cranmer Terrace London SW17 ORE UK


**Comparative and Functional Genomics**
*Comp Funct Genom* 2002; **3**: 326–329.
Published online in Wiley InterScience (www.interscience.wiley.com). **DOI:** 10.1002/cfg.189

**Conference Editorial**

### The 1st Symposium of The Wellcome Trust-funded Multi-collaborative Microbial Pathogen Microarray Facility — BμG@S 2002: 'Bacterial pathogens, microarrays and functional genomics'

The Wellcome Trust Genome Campus, Hinxton Hall, Cambridge, UK, 23–24 May 2002

Philip D. Butcher*
*Department of Medical Microbiology, St George's Hospital Medical School, Cranmer Terrace, London SW17 ORE, UK*

**Correspondence to:
Philip D. Butcher,
Department of Medical Microbiology, St George's Hospital Medical School,
Cranmer Terrace,
London SW17 ORE, UK.
E-mail: butcherp@sghms.ac.uk*

Received: 11 June 2002
Accepted: 14 June 2002

### Overview

The acquisition of increasing numbers of bacterial whole-genome sequences (>50) has coincided with the development of a range of technologies to study the function of genes on a genome-wide scale, which are grouped under the broad heading of functional genomics. In terms of bacterial pathogenesis, one of the current major research interests lies in defining the complement of genes that determine virulence (the virulome) and how these genes may be coordinately regulated (the regulome) and expressed at the level of mRNA (the transcriptome) and protein (the proteome). Bacterial virulence may be defined as the appropriate temporal and spatial expression of overlapping subsets of genes necessary for a particular phase of infection, in response to specific environmental signals encountered within the host.

A rapidly growing literature using microarrays for bacterial functional genomics has confirmed the reality and robust nature of spotted DNA glass-slide microarrays for small genome organisms. The relative biological simplicity of bacterial genomes and thus the straightforward nature of the design and construction of whole genome microarrays facilitates the analysis of bacterial whole genomes by both comparative genomics and gene expression profiling. Together they provide a powerful approach to the definition of genes important in pathogenesis and offer the prospect of useful targets for rational design of new drugs and vaccine candidates for bacterial pathogens.

Post-genome biology divides into observational biology to drive hypotheses, and experimental genomics that use some of the new tools to test hypotheses. Microarrays will potentially deliver both. There is a prominent place for

Copyright © 2002 John Wiley & Sons, Ltd.

hypothesis-generating (observational) experiments in genome biology, which implicitly test our current understanding of gene expression, regulation and function. This BμG@S 2002 meeting on bacterial microarrays showed the value of both approaches in comparative and functional genomics of bacteria. In this new era of functional genomics, scientists of different disciplines and skills are increasingly being asked to work together — molecular biologists, cell biologists, clinical scientists, computer scientists, statisticians, mathematicians and engineers. The meeting illustrated the benefits of such interdisciplinary collaborations and highlighted the progress made by a number of academic groups around the UK studying bacterial pathogens using whole-genome microarrays and accessing newly developed mathematical and statistical data analysis tools. Despite the relative simplicity of the organism, the task faced by bacteriologists is immense. The ability to study every bacterial strain, or monitor expression profiles with time under every condition is clearly neither a practical proposition nor a well-considered scientific goal. With limited resources and finances more focused experiments are required. This necessitates collaborative efforts and data comparisons from different experiments, e.g. DNA damage responses, the SOS response, stress responses and *in vivo* expression profiles, such as during macrophage exposure, or conditions that may mimic some aspect of the host environment. Common-format databases for data comparison will accelerate and facilitate this process and are being established (Witney and Hinds, p. 369).

### BμG@S 2002

The general aim of the BμG@S 2002 meeting was to present the results of experiments using bacterial microarrays made by a multi-collaborative and multi-disciplinary research group: the microbial pathogen microarray facility (BμG@S) based at St George's Hospital Medical School, London. BμG@S was funded by The Wellcome Trust in 2001 under the 'Resources for Functional Genomics Initiative'. Its main aim is to construct whole-genome arrays for 12 bacterial pathogens in 2 years (2001–2003) and to make them available over a 5 year period to an extensive network of collaborative academic research groups around the UK (Hinds *et al.*, p. 333; see also **http://bugs.sghms.ac.uk**). Microarrays for *Mycobacterium tuberculosis, Campylobacter jejuni, Haemophilus influenzae, Streptococcus pneumoniae* and *Yersinia pestis* are already in use at BμG@S and most of the presentations at the meeting therefore related to these pathogens. Many of the topics presented at the meeting are covered in this supplement as conference reviews on a subject/pathogen basis.

There were over 150 delegates invited from the collaborating groups. The meeting opened with an introduction about the collaborative ethos of BμG@S and how this unique microarray resource was established (**Dr Philip Butcher, St George's Hospital Medical School, London**). A review of the potential of microarrays in bacterial functional genomics was then presented by **Professor Brendan Wren (London School of Hygiene and Tropical Medicine)** in which the latest 'headline' findings in bacterial pathogenicity were explored (Wren, p. 330). This was followed by an overview of the multi-collaborative network of BμG@S and its progress with making and utilizing the microarrays (Hinds *et al.*, p. 333). The keynote speaker was **Dr Rob Fleischmann** from the **Institute of Genomic Research (TIGR), USA**, who talked about the NIAID-sponsored Pathogen Functional Genomics Resource Centre at TIGR, and also presented research results on the *Streptococcus pneumoniae* competence system.

The meeting's subject matter was structured into three main areas: comparative genotyping, gene expression and data analysis (see below). A fourth and final session consisted of contributions from representatives of other UK microbial genomics resources (**Dr Al Ivens** from the **Sanger Institute Pathogen Sequencing Unit** and **Dr Tom Freeman** from the **MRC HGMP-RC**), who talked about the genomic resources available to the academic community. Also, other collaborating academic research groups that make and use microbial microarrays presented their research and described their microarray facilities. This included **Dr Colin Smith (UMIST, Manchester)**, who talked about the *Streptomyces* microarray and presented cluster analysis of gene expression data during developmental and metabolic transitions in *Streptomyces.* **Dr Jay Hinton** (**Institute of Food Research, Norwich**) presented array data to show that point mutations in polynucleotide phosphorylase (PNPase) of

Copyright © 2002 John Wiley & Sons, Ltd. 

*Salmonella enterica* affected the expression of subsets of genes, including the SPI1 and SPI2 virulence genes, indicating a role for PNPase in a novel form of global virulence gene regulation.

### Comparative genomics

Microarray experiments broadly divide into those in which genomic DNA is used in hybridizations, and expression studies using RNA-derived cDNA. Not surprisingly, due to simpler analysis, the former has advanced most quickly. The comparative genomics section of the meeting highlighted the use of arrays in *Mycobacteria, Yersinia* and *Campylobacter* species. Subjects covered included bacterial taxonomy, genome indexing and phenotypic correlation in clinical isolates for virulence gene mapping. A well-studied example was the partially overlapping plasmid-based whole-genome *C. jejuni* microarray, which revealed extensive genetic diversity amongst clinical strains, with at least 21% of genes being dispensable. These genes were predominantly associated with the biosynthesis of surface structures, including flagella, lipooligosaccharide and the capsule, as well as those responsible for iron acquisition, DNA restriction and sialylation (Dorrell *et al*., p. 338). Also of particular note were the immediate public health applications of bacterial arrays in human (*M. tuberculosis*) and animal (*M. bovis*) pathogen molecular epidemiological studies and in community outbreak investigations (respectively, Inwald *et al*., p. 342 and Shafi *et al*., p. 362).

Clearly, arrays are powerful tools but do not comprehensively probe entire genomes of all new strains, since arrays contain elements (spotted DNA) from selected sequenced 'reference' strains and therefore only detect deleted genes in the test strains. Without whole-genome sequencing of every isolate, microarrays are thus limited to known sequenced genes. Constructing pan-genus or pan-species arrays representative of all sequenced genes in different strains or species on one array goes some way towards meeting such limitations and was highlighted as a priority for BμG@S (Wren, p. 330). A major challenge, therefore, with bacterial genomes is to define gene additions; this may be accomplished by sequencing subtractive hybridization products between references and test strains for subsequent inclusion on arrays. Another approach might be to assume that deletion sites detected on arrays might also be sites for insertions; PCR amplification across deletions therefore may reveal inserted sequences.

### Gene expression

Gene expression studies are more difficult to perform and analyse than comparative DNA hybridizations. The variation introduced into such experiments, which was fully explored in this meeting, derives from biological as well as experimental variation. Subtle differences in bacterial growth rate, cell density and environmental conditions may influence gene expression profiles. Emphasis was placed on the extraction of 'biologically meaningful' RNA and the avoidance of mRNA expression artefacts due to sample preparation. A range of talks on gene expression profiling during various *in vitro* conditions were presented (see conference reviews). Significantly, the use of chemostat cultures of *M. tuberculosis* (James *et al*., p. 345) showed the value of such systems in reducing the biological variability in complex heterogeneous batch cultures of bacteria and also in permitting a more robust statistical analysis of the data produced. Examples of microarrays to dissect the bacterial regulome and functionally analyse promoter elements were presented by the use of transcriptional regulatory mutants in *M. tuberculosis* (Kendall *et al*., p. 352 and Stewart *et al*., p. 348), the response regulator *pnpR* of *Streptococcus pneumoniae* (McCluskey *et al*., p. 366) and mutants of alternative sigma factors *fliA* and *rpoN* in *C. jejuni* (Dorrell *et al*., p. 338).

Other issues relating to expression data were raised during the meeting. These included the ability to overlay expression data onto both genome structure and metabolic pathways, so as to be able to experimentally confirm operon organization or deduce the use of certain metabolic pathways, respectively. Also discussed was the issue that small changes in mRNA may produce big changes in phenotype, and that the often-used two-fold change cut-off values should be viewed as arbitrary levels for ease of data handling, and not as the value above which biological significance may be inferred. Although it is reported increasingly in the literature that fold-changes in mRNA of 1.5–1.9 can be statistically measured, such

Copyright © 2002 John Wiley & Sons, Ltd. 

accuracy remains a challenge for most microarray experiments. However, without accurate measurements at such levels, much biological information will be lost. A key feature of discussion from the transcriptome work presented was whether microarray data required validation by other methods, such as RT–PCR. Clearly, not all genes can be validated in this way, but target genes for further studies (such as making mutants) should be tested, despite the good correlation that is now accepted between microarray data and quantitative RT–PCR or Northern blotting. Good statistical analysis of array data is of primary importance, but this is limited by biological and experimental variation and the number of replicates: 'One array does not a summer make!'.

### Data analysis

Such considerations led on to the part of the meeting on data analysis, where variation in data and how to extract meaningful information were the main themes. Variation in microarray experiments exists at different levels: biological, experimental, analytical (image and data analysis) and interpretative. Experimental design from the statistical view and the need to randomize both experimental set-up and the microarray format itself, were considered important. Ways to optimize the number of replicates (biological and experimental) required for robust statistical validity of data (Wernisch, p. 372) and approaches to data visualization and mathematical modelling of array data sets (Wolkenhauer *et al.*, p. 375) were also presented. Well-designed microarray experiments require close and continued association of statistician, biologist and mathematician. This meeting exemplified such a collaborative approach to bacterial functional genomics and exposed data analysis as a major bottleneck. In the context of facilitating microarray data handling and interrogation, a microarray relational database created by Dr Adam Witney from BμG@S was demonstrated. This database, called BμG@Sbase, enables all aspects of the construction of a particular array to be viewed from gene sequence, PCR primer pairs, PCR product gel electrophoresis, to array format with spot identity enquiry (Witney and Hinds, p. 369). Development of the database to include experimental data and integrate analysis tools will ultimately enable the full repertoire of microarray data handling and analysis to be accomplished from a single computer format.

### Summary

Perhaps the most significant feature of the meeting was the fact that so many people were describing experiments they had done, and the significance of the results, as well as the problems they had encountered. This contrasts with many meetings to date, where talks have mostly discussed array production and experiments that are planned. However, microarray experimental systems are an emerging and rapidly developing technology and thus still have limitations and pitfalls. This meeting set out to explore such limitations in the hope that sharing experiences in a collaborative environment would allow collective progress towards development of robust technical protocols and data analysis tools. Despite limitations, widespread availability and use of DNA microarrays in the current format for small genomes are now of undisputed biological utility. BμG@S is an example of a functional genomics resource for multi-collaborative networks of research groups and can act as a model for functional genomics initiatives for more complex organisms (e.g. humans). The meeting showed how microarray technology can address both fundamental and applied issues in pathogen biology: from comparative genome organization through to studies on pathogenesis, virulence genes, molecular epidemiology and public health. We should now look forward to exploiting these microarray resources and expanding the multi-disciplinary institutional and personal collaborations necessary for rapid progress in bacterial functional genomics.

### Acknowledgements

The Wellcome Trust is acknowledged for financial support to BμG@S and for a conference grant. Highly productive technical partnerships with BioRobotics Ltd and MWG Biotech Ltd are also acknowledged. The meeting was, in part, co-sponsored by the following commercial companies: BioRobotics, Invitrogen, MWG Biotech, Qiagen, Sigma-Aldrich, Silicon Genetics and Stratagene.

Copyright © 2002 John Wiley & Sons, Ltd.

